# The use of ozone gas for the inactivation of *Bacillus anthracis* and *Bacillus subtilis* spores on building materials

**DOI:** 10.1371/journal.pone.0233291

**Published:** 2020-05-21

**Authors:** Joseph P. Wood, Morgan Wendling, William Richter, James Rogers

**Affiliations:** 1 Office of Research and Development, U.S. Environmental Protection Agency, National Homeland Security Research Program, Research Triangle Park, North Carolina, United States of America; 2 Battelle Memorial Institute, Columbus, Ohio, United States of America; University of Connecticut, UNITED STATES

## Abstract

A study was conducted to assess the efficacy of ozone gas in inactivating spores of both *Bacillus anthracis* and *Bacillus subtilis* inoculated onto six building materials (glass, wood, carpet, laminate, galvanized metal, and wallboard paper). Testing conditions consisted of ozone gas concentrations ranging from 7,000–12,000 parts per million (ppm), contact times from 4 to 12 h, and two relative humidity (RH) levels of 75 and 85%. Results showed that increasing the ozone concentration, contact time, and RH generally increased decontamination efficacy. The materials in which the highest decontamination efficacy was achieved for *B*. *anthracis* spores were wallboard paper, carpet, and wood with ≥ 6 log_10_ reduction (LR) occurring with 9,800 ppm ozone, 85% RH, for 6 h. The laminate and galvanized metal materials were generally more difficult to decontaminate, requiring 12,000 ppm ozone, 85% RH, and 9–12 h contact time to achieve ≥6 LR of *B*. *anthracis*. Lastly, overall, there were no significant differences in decontamination efficacy between the two species.

## Introduction

The recently published 2018 National Biodefense Strategy calls for the U.S. to manage the risk of biological incidents and, specifically, conduct research and development of technologies to enhance preparedness to support decontamination. These biological threats can be categorized as naturally occurring, accidental, or deliberate attacks[1], such as the dissemination of virulent spores of *Bacillus anthracis* through the U.S. Postal Service[2]. Since this 2001 attack, a large body of research and development has been undertaken to evaluate decontamination techniques for this bioterrorism agent, and is reviewed here[3]. The present study builds on that research and supports the National Biodefense Strategy by verifying the efficacy of ozone gas as a decontaminant for inactivating *B*. *anthracis* spores deposited on various types of building materials.

The use of ozone gas for the inactivation of vegetative and spore-forming microorganisms on surfaces has been explored since at least 1982 [4]. More recently, ozone gas has been tested and is gaining traction for use in healthcare environments[5] for disinfection (in particular, inactivation of vegetative bacteria[6] and viruses[7]) and sterilization (inactivation of spore-forming microorganisms such as *B*. *anthracis*, *Clostridioides difficile*[8], and *Bacillus cereus*[6]). In addition, ozone gas has been approved for reprocessing (sterilization) of medical equipment that cannot be heat-treated [9]. The mechanism for the killing of spores by ozone is thought to be through damage to the inner membrane, thereby rendering the spores defective in germination[10]. The research described herein did not further investigate or confirm these mechanisms, but rather strived to elucidate the environmental and operational conditions necessary for inactivation of *B*. *anthracis* spores with ozone gas. Using ozone gas at relatively low concentrations (1–25 parts per million [ppm]), Akbas et al.[11] and Sharma et al. [6] demonstrated moderate efficacy (2–4 log_10_ reduction [LR]) against *B*. *cereus* and *C*. *difficile* spores on a number of different materials including figs, fabrics, and plastics. At approximately 5,000 ppm, 90% relative humidity (RH), and 22 °C for 4 h, Aydogan et al.[12] also reported only a moderate 2–4 LR (depending on the interior building material) in viable *B*. *subtilis* spore populations. An ozone concentration of 9,000 ppm ozone in air and a 1 h contact time were required to achieve > 6 LR of *Bacillus atrophaeus* (aka *Bacillus globigii*) spores on glass slides, with at least 70% RH and exposing spores to at least 70% RH levels for 15 h prior to exposure to ozone[13]. Mahfoudh et al. [14] discuss the role that elevated RH plays in fumigation with ozone and other gaseous decontaminants, via the swelling of spores and creating “channels” for gas to diffuse into the spore. Other research groups [12, 15, 16] have corroborated the finding that increasing RH levels (> 80%) improves efficacy with ozone gas. Indeed, a higher RH during fumigation with other gases[3] or with hot air [17–20] is usually associated with improved decontamination efficacy for *B*. *anthracis* spores.

While the literature mentioned above contributes to the science and engineering of using ozone gas as a sporicide, there are few data available showing ozone fumigation conditions required for achieving ≥ 6 LR of a spore population. The “6 LR” benchmark originates from efficacy testing guidance for antimicrobial chemicals with claims to inactivate spores of *B*. *anthracis* on surfaces[21]. Additionally, there is a paucity of data for the use of ozone gas in the inactivation of *B*. *anthracis* spores on surfaces. In a short conference proceeding, Buranov et al.[22] indicated that 20 mg/L (~ 10,000 ppm) ozone for 1 h was sufficient for complete inactivation of 10^6^ and 10^8^ spores of a *B*. *anthracis* vaccine strain deposited on an empty Petri plate; however, minimal data and details on the methods used were provided.

The present study fills these research gaps by presenting data for the first time verifying the conditions necessary (required ozone gas concentrations, RH levels) for effective inactivation (i.e., ≥ 6 LR) of virulent *B*. *anthracis* (Ames strain) spores using ozone gas. In addition, decontamination efficacy data are presented for the first time for both *B*. *subtilis* and *B*. *anthracis* tested under the same conditions to allow comparison of the resistance to ozone gas for the two species and verify use of *B*. *subtilis* as a potential surrogate for *B*. *anthracis* when conducting decontamination tests with ozone gas. *B*. *subtilis* was chosen for comparison with *B*. *anthracis*, consistent with its demonstration as a suitable surrogate in previous other decontamination studies[23–26]. Although we do acknowledge that the use of *Bacillus thuringiensis* as a surrogate for *B*. *anthracis* in decontamination studies is becoming more widely used [17–19, 27]. Lastly, the present study is novel for evaluating decontamination efficacy with *B*. *anthracis* spores inoculated on common realistic interior and exterior building materials. Many biological decontamination studies tend to use only one or two basic laboratory substrates such as glass or Petri plates and overlook the typically significant effect that material has on efficacy.

## Materials and methods

### Microorganisms

Testing was conducted with *B*. *anthracis* (Ames strain) spores produced at Battelle Biomedical Research Center in West Jefferson, OH, and verified via genotyping. The virulent Ames strain used in this study was the strain used in the 2001 *B*. *anthracis* letter attacks.[28] All portions of this testing were performed under Biosafety Level 3 conditions, in accordance with the Federal Select Agent Program (FSAP) regulations set forth by the Centers for Disease Control and Prevention.

Testing was also conducted with *B*. *subtilis* spores (ATCC 19659) from a stock culture. Details of the methods used to produce spores of both species are published elsewhere in several previous articles[24, 26, 29, 30]. Briefly, the *B*. *anthracis* spores were prepared using a BioFlo 3000 fermentor (New Brunswick Scientific Co., Inc., Edison, NJ, USA). A culture was grown for 16–18 h at 37 °C in nutrient broth (BD Diagnostic Systems, Sparks, MD, USA), and was then used to inoculate Leighton-Doi Broth (BD Diagnostic Systems) in the fermentor. Cultures were grown in the fermentor for 24 h at 37 °C. Cultures exhibiting >80% refractile spores via phase-contrast microscopy were centrifuged at 11,000 g for 15–20 min at 2–8 °C. The resultant pellet was washed twice and resuspended in ice-cold sterile water to further remove cellular debris. To inactivate any vegetative cells, the suspension was then heat-shocked by incubating at 60 °C for 45–60 min, then centrifuged and washed again. Final preparation involved centrifuging the sample through a gradient of ice-cold, sterile 58% Hypaque-76 (Nycomed Amersham, Princeton, NJ, USA) at 9,000 g for 2 h at 2–8 °C. This batch of spores was used throughout the study. *B*. *subtilis* stock suspensions were prepared in a manner similar to the above procedures for *B*. *anthracis* and as previously described [31]. The resultant *B*. *anthracis* and *B*. *subtilis* spore preparations having >95% refractile spores with <5% cellular debris were enumerated, diluted in sterile water to a target of 10^9^ colony-forming units (CFU) per mL, and stored at 2–8 °C. Spores were also tested to ensure HCl resistance for 2 min. No specific tests were performed to check for germination while the spores were stored at 2–8 °C, however a check on the titer of the spore lot was performed at the start of each test. This was to ensure that the titer of the stock was not changing, providing an indication that no germination was happening.

### Coupon experimental materials

Decontamination testing was conducted using realistic building exterior and interior materials. These materials included glass, wood, carpet, laminate, galvanized metal, and painted wallboard paper. Information on the materials and associated sterilization approaches is presented in Table 1. Coupons of the materials were cut to 1.9 cm width by 7.5 cm length from larger pieces. Coupons were then sterilized by autoclaving or gamma irradiation (at ~40 kilogray), depending on cost and compatibility with materials.

**Table 1:** Information on materials used for test coupons.

Material	Lot/Batch/Observation	Manufacturer/Supplier Name	Material Preparation
Glass	C1036	Brooks Brothers Glass Columbus, OH	Autoclave
Wood (untreated pine)	Generic modeling	West Jefferson Hardware West Jefferson, OH	Gamma irradiation
Carpet	Shaw EcoTek 6	Grossmans Bargain Outlet; Columbus, OH	Gamma irradiation
Laminate	NA	A’Jack Inc. Columbus, OH	Gamma irradiation
Metal ductwork (galvanized metal)	NA	Adept Products West Jefferson, OH	Autoclave
Painted wallboard Paper	05-16-03; Set-E-493; Roll-3	United States Gypsum Company; Chicago, IL	Gamma irradiation

### Inoculation, recovery, and quantification of microorganisms

Each of the test and positive control coupons was inoculated with a target of 1 x 10^8^ CFU of either *B*. *anthracis* or *B*. *subtilis* spores. (The spore suspension titer was verified each day of testing.) A 100-μL aliquot of a stock suspension of approximately 1 x 10^9^ CFU/mL, dispensed using a micropipette, was applied as 10 μL droplets across the coupon surface. After inoculation, the coupons were placed in a biosafety cabinet (BSC) to allow the suspension to dry overnight, under laboratory ambient conditions of approximately 22 °C and 40% RH. The number and type of replicate coupons used for each combination of microorganism, material, and test condition (ozone concentration, RH, and contact time) were as follows:

- 5 replicate test coupons (inoculated with *B*. *anthracis* or *B*. *subtilis* spores and exposed to ozone)
- 5 positive controls (inoculated with *B*. *anthracis* or *B*. *subtilis* spores, but not exposed to ozone)
- 1 laboratory blank (inoculated only with sterile water and not exposed to ozone)
- 1 procedural blank (inoculated only with sterile water and exposed to ozone for the longest contact time).

Following each test, spores were extracted from each coupon as follows: Each coupon (test coupons, positive controls, and blanks) was placed in a 50-mL polypropylene conical vial containing 10 mL of sterile phosphate-buffered saline extraction buffer containing 0.1% Triton X-100 surfactant (Sigma, St. Louis, MO, USA). The vials were capped, placed on their side and agitated on an orbital shaker for 15 minutes at approximately 200 revolutions per minute (rpm) at room temperature. Spores were then quantified from this extraction suspension using standard dilution plating techniques. Briefly, the extract was removed, and a series of 10-fold dilutions was prepared in sterile water. An aliquot (0.1 mL) of either the undiluted extract and/or each serial dilution was plated onto tryptic soy agar in triplicate. The cultures were incubated for 18–24 h at 37 °C ± 2 °C for *B*. *anthracis* and 35 °C ± 2 °C for *B*. *subtilis*. Colonies were counted manually, and the CFU/mL value was determined by multiplying the average number of colonies per plate by the reciprocal of the dilution. Dilution data representing the greatest number of individually definable colonies (within a range of 25–250 discernible colonies per plate) were expressed as arithmetic mean ± standard deviation of the numbers of CFU observed. The CFU recovered from each coupon were determined by multiplying the CFU/mL by 10 (as 10 mL was used to extract each coupon).

### Ozone gas concentration, temperature, and RH production and measurement

Ozone gas exposure testing was conducted inside a Class III Biological Safety cabinet (The Baker Company, Sanford, ME, USA) (0.57 m^3^). Ozone gas used in the test chamber was produced with a commercially-available generator (AC-2045, IN USA Inc., Norwood, MA, USA), which used 99.5% oxygen as its feedstock. The generator was run at 100% power until the target concentration in the test chamber was reached, and subsequently reduced to approximately 30% power to maintain concentration. Ozone gas concentrations < 10,000 ppm or > 10,000 ppm were measured with a low (IN-2000-L2-LC, IN USA Inc., Needham, MA, USA) or high (Mini Hicon-LR, IN USA Inc.) concentration gas analyzer, respectively. Both ozone gas analyzers were calibrated by the manufacturer prior to shipment. Ozone concentrations were logged automatically every minute using a HOBO data logger (Onset Computer Corporation, Bourne, MA, USA).

A custom-made ultrasonic fogger was used to raise humidity inside the test chamber. Air saturated with moisture from the fogger was injected into the chamber via polyvinyl chloride tubing until the target RH was reached. Additional humidified air was injected into the chamber as needed to maintain target RH levels. In initial method development tests, an off-the-shelf hygrometer was used to measure RH, but the hygrometer became readily damaged upon exposure to ozone gas. Hence, a wet/dry bulb hygrometer was custom-fabricated using two National Institute of Standards and Technology [NIST]-traceable thermometers [Fisher 13-990-270]) and used to monitor the temperature and RH inside the test chamber. For the wet bulb thermometer, a wicking material was wrapped around one thermometer bulb, and the wick was kept wet by immersion in a small reservoir of water. The other thermometer provided the dry temperature reading. A small fan (Cooler Guys, UF12BWL, Kirkland, WA, USA) provided constant air flow for the thermometers and for mixing inside the chamber. Differences in the dry and wet bulb temperature readings (taken and manually recorded every 2–15 minutes) were used to determine RH, using an online humidity calculator [32].

For each experiment (one ozone concentration and RH level), coupons inoculated with *B*. *anthracis* or *B*. *subtilis* spores were placed in sealed containers (Lock & Lock, HPL838P, Farmers Branch, TX, USA) and then placed into the test chamber. All containers were open until the target RH was achieved. Then, all containers were closed during the initial injection of ozone. The containers holding the test coupons were then opened sequentially to achieve an appropriate contact time for exposure to ozone. Contact time was defined as the time from opening the container to the time ozone was exhausted from the chamber. Positive controls and laboratory blanks were also kept in sealed containers for the full fumigation cycle. The procedural blank coupons were opened for exposure to ozone at the same time as the test coupons with the longest contact time (e.g., 8 or 12 h). At the end of the fumigation cycle, the ozone generator was shut down and the ozone rapidly exhausted until the ozone analyzer read zero. All test coupons were subsequently removed from the BSC III and processed for remaining viable spores.

### Test matrix and ozone fumigation procedures

The overall test matrix for the study is shown in Table 2. Test variables for the study included microorganism, building material, ozone concentration, contact time, and RH level. Ozone concentrations ranged from 7,000–12,000 ppm. Tests for each microorganism (*B*. *subtilis*, *B*. *anthracis* Ames) were conducted separately but under the same conditions, and all six materials were used in every experiment. Three contact times were evaluated for each of the seven fumigation conditions (ozone concentration and RH level). At the 9,000-ppm concentration, the 85% RH condition was tested twice to evaluate longer ozone exposure times. At the lower concentrations of 7,000 and 9,000 ppm ozone, efficacy at both 75 and 85% RH was evaluated to assess the effect of RH. To ensure demonstration of effective decontamination for all materials, the remaining experiments were designed to focus on higher ozone concentrations at 85% RH for contact times out to 12 h.

**Table 2:** Study test matrix.

Test number	Target Ozone Concentration, ppm	% RH	Contact Time, h
1	7,000	85	4, 6, 8
2	7,000	75	4, 6, 8
3	9,000	85	4, 6, 8
4	9,000	75	4, 6, 8
5	9,000	85	6, 9, 12
6	9,800	85	6, 9, 12
7	12,000	85	6, 9, 12

All experiments performed at ~25 °C

### Calculation of spore recovery and decontamination efficacy

The methods we used for determining spore recovery and decontamination efficacy are described elsewhere.[30] Briefly, the average percent spore recovery from each set of positive controls for a given material was calculated for each test and microorganism using the following equation:

$$\text{Mean}\;\%\;\text{Recovery}=[\text{Mean}\;{\text{CFU}}_{\text{pc}}/{\text{CFU}}_{\text{spike}}]\times 100$$ (1)

where Mean CFU_pc_ is the mean number of CFU recovered from the five replicate positive control (pc) coupons for a given material, and CFU_spike_ is the number of CFU spiked (inoculated) onto each of those coupons, determined via analysis of the inoculum each day of testing.

Decontamination efficacy was calculated in terms of LR for each material, microbe, and test condition. For each test (noted with subscript t) and positive control coupon, the number of CFU recovered after each test was transformed to its log_10_ value. Then, the mean of the log_10_ values for each test coupon (five replicates) was subtracted from the mean of the log_10_ values from each positive control (five replicates), as follows:

$$Efficacy=\bar{\left(log\;CFUpc\right)}-\bar{\left(log\;CFUt\right)}$$ (2)

Test coupons in which there were no CFU recovered were assigned a CFU count of 1, resulting in a log_10_ CFU of zero. In such cases, the LR is reported as ≥ the value calculated by Eq 2.

The LR results are each reported with an associated 95% confidence interval (CI), calculated as follows:

95%CI=Efficacy±(1.96×SE) (3)

The term SE is the pooled standard error, and was calculated as follows:

$$SE=\sqrt{\frac{S^{\text{2}}pc}{\text{5}}+\frac{S^{2}t}{5}}$$ (4)

where S is the standard deviation of the LR results for either the five positive controls (pcs) or five test coupons (t) for each combination of decontaminant, coupon material, and microorganism tested.

### Statistical analyses

A multilevel model was performed that included microorganism, material, RH, and ozone dosage (ozone concentration × exposure time) as predictors of efficacy. To account for the nonlinearity observed in the response, ozone concentration and exposure time were additionally included in the model as factors. Validity conditions were assessed using diagnostic plots and/or statistical tests (where appropriate) to assist in justifying the approach taken. When fitting a multilevel model, it is assumed that there is a linear relationship between the predictors and the response, the random errors are homogeneous, the errors are uncorrelated, and the error terms are normally distributed. F-tests were performed on the model to help address the questions of interest for main effects and interactions investigating if there are any differences, statistically, between levels or values of predictors. We then performed t-tests on the model to explore in more depth how the response differs across levels or values of predictors. There were interactions present among the predictors, and we determined how the response differs across levels or whether the values of one predictor are conditional on other predictors. All statistical analyses were performed in R (https://www.r-project.org/).

In addition to the statistical analysis, a simple ranking approach was sometimes used to distinguish the effect of test variables (such as material, RH) on efficacy, i.e., some results were ranked or described in terms of the number of occurrences in which the decontamination was effective. We consider a decontamination result “effective” if the efficacy is ≥ 6 LR, which is a benchmark based on guidance provided for the evaluation of antimicrobial products with claims to inactivate *B*. *anthracis* spores on surfaces [21]. Another criterion used to rank efficacy results was based on whether the decontamination treatment or test condition resulted in no spores recovered from all five replicate test coupons following decontamination; this condition is sometimes referred to as complete inactivation or complete decontamination.

## Results and discussion

### Fumigation conditions

Actual fumigation conditions, in terms of the average temperature, RH, and ozone concentration for each experiment, did not deviate significantly from the target conditions. That is, with a target temperature of 25 °C for all tests, actual average temperatures ranged from 24.2–26.0 °C. For RH, actual levels typically did not deviate more than two percentage points from the target levels of either 75 or 85%. For ozone concentration, average actual levels deviated no more than 28 ppm from the targets. The actual fumigation conditions for each experiment are summarized in Tables 3 and 4.

**Table 3:** Summary of actual temperature, relative humidity and ozone conditions for *B*. *anthracis* tests.

Test Number	Target Test Conditions, Ozone %RH^+^	Contact Time h	Temperature °C*	%RH*	Ozone ppm*
1	7,000 ppm 85 ± 5%	4	25.3 ± 0.5	90.0 ± 0.5	7,000 ± 40
		6	25.4 ± 0.5	89.6 ± 0.9	7,021 ± 71
		8	25.2 ± 0.6	88.4 ± 2.4	7,021 ± 102
2	7,000 ppm 75 ± 5%	4	24.4 ± 0.4	76.9 ± 2.8	7,007 ± 35
		6	24.5 ± 0.4	76.3 ± 2.9	7,011 ± 43
		8	24.5 ± 0.4	76.3 ± 2.6	7,011 ± 39
3	9,000 ppm 85 ± 5%	4	24.7 ± 0.5	85.6 ± 1.7	9,004 ± 43
		6	24.7 ± 0.5	84.9 ± 1.9	9,002 ± 44
		8	24.6 ± 0.5	85.0 ± 1.8	9,003 ± 44
4	9,000 ppm 75 ± 5%	4	24.4 ± 0.7	75.8 ± 2.0	9,007 ± 68
		6	24.3 ± 0.6	75.9 ± 1.8	9,015 ± 75
		8	24.2 ± 0.6	76.4 ± 2.0	9,017 ± 75
5	9,000 ppm 85 ± 5%	6	25.8 ± 0.5	85.9 ± 1.6	8,996 ± 32
		9	25.7 ± 0.5	85.5 ± 1.6	8,995 ± 40
		12	25.4 ± 0.8	85.4 ± 1.6	8,982 ± 73
6	9,800 ppm 85 ± 5%	6	25.9 ± 0.5	85.2 ± 1.0	9,801 ± 55
		9	25.9 ± 0.5	85.3 ± 1.0	9,798 ± 54
		12	25.8 ± 0.6	85.2 ± 1.1	9,798 ± 52
7	12,000 ppm 85 ± 5%	6	24.8 ± 0.5	84.4 ± 1.8	11,982 ± 63
		9	24.8 ± 0.5	84.7 ± 2.3	11,972 ± 73
		12	24.6 ± 0.6	84.7 ± 2.4	11,981 ± 68

*Data are presented as mean ± standard deviation.

**Table 4:** Summary of actual temperature, relative humidity and ozone conditions for *B*. *subtilis* tests.

Test Number	Target Test Conditions, Ozone %RH^+^	Contact Time h	Dry Bulb Thermometer, °C*	%RH*	Ozone ppm*
1	7,000 ppm 85 ± 5%	4	25.6 ± 0.7	86.2 ± 1.8	6,998 ± 32
		6	25.6 ± 0.6	86.4 ± 2.3	7,002 ± 36
		8	25.6 ± 0.6	86.2 ± 2.4	7,008 ± 51
2	7,000 ppm 75 ± 5%	4	24.4 ± 0.4	75.5 ± 1.9	7,003 ± 18
		6	24.5 ± 0.5	75.5 ± 2.0	6,996 ± 33
		8	24.5 ± 0.5	75.7 ± 1.9	6,996 ± 29
3	9,000 ppm 85 ± 5%	4	24.3 ± 0.5	84.2 ± 1.8	9,007 ± 33
		6	24.4 ± 0.5	84.6 ± 1.7	9,006 ± 35
		8	24.3 ± 0.5	85.2 ± 2.1	9,003 ± 35
4	9,000 ppm 75 ± 5%	4	24.7 ± 0.4	76.0 ± 2.2	9,011 ± 48
		6	24.7 ± 0.5	76.1 ± 2.4	9,011 ± 48
		8	24.5 ± 0.6	76.0 ± 2.3	9,003 ± 43
5	9,000 85 ± 5%	6	26.0 ± 0.4	85.1 ± 0.8	9,010 ± 44
		9	25.8 ± 0.5	85.0 ± 0.9	9,009 ± 40
		12	25.6 ± 0.7	84.8 ± 1.1	9,009 ± 38
6	9,800 ppm 85 ± 5%	6	25.6 ± 0.5	85.1 ± 1.9	9,791 ± 39
		9	25.7 ± 0.5	85.4 ± 2.0	9,786 ± 39
		12	25.6 ± 0.6	85.1 ± 2.0	9,785 ± 43
7	12,000 ppm 85 ± 5%	6	24.8 ± 0.4	85.2 ± 1.5	12,009 ± 106
		9	24.7 ± 0.4	84.9 ± 1.4	11,999 ± 101
		12	24.6 ± 0.6	85.1 ± 1.7	12,008 ± 94

* Data are presented as mean ± standard deviation.

### Inoculation and recovery of spores from positive controls

For the overall study, the average (± SD) number of *B*. *anthracis* spores inoculated onto each coupon in each trial was 8.01 ± 0.08 log CFU and for *B*. *subtilis*, the number of spores was 8.05 ± 0.21 log CFU. The average percent recovery for each microorganism from the positive controls is tabulated in Table 5 for each material type. The mean percent recovery from positive controls was higher for the *B*. *anthracis* compared to *B*. *subtilis*, for five of the six materials, although the estimated differences in percent recovery were relatively minor. Overall, the average percent spore recoveries ranged from 3%-77%. The lowest percent recoveries for both species were obtained for wood positive controls and averaged less than 10%. Nevertheless, recoveries for both species from wood positive controls were always greater than 6 log CFU, allowing the decontamination condition to demonstrate ≥ 6 LR. For *B*. *subtilis*, the highest recoveries were obtained for galvanized metal, and for *B*. *anthracis*, highest recoveries were obtained with carpet. These results for percent recovery of spores from the positive control materials are similar and consistent with other decontamination studies using spores of *B*. *anthracis* and *B*. *subtilis* [26, 33].

**Table 5:** Average percent recovery ± SD of each microorganism from positive controls.

	*B*. *anthracis*	*B*. *subtilis*
Glass	56 ± 20	38 ± 9
Wood	8 ± 5	3 ± 1
Carpet	77 ± 16	45 ± 7
Laminate	45 ± 12	51 ± 12
Galvanized metal	55 ± 9	54 ± 16
Wallboard paper	50 ± 30	8 ± 5

n = 35 for each material/microorganism (seven experiments × five positive controls per experiment)

### Decontamination efficacy of ozone gas

#### Overview/effect of concentration

The decontamination efficacy results, in terms of average LR, are summarized in Tables 6 and 7 for *B*. *anthracis* spores and *B*. *subtilis* spores, respectively (Also refer to the Supporting Information S1 File, S1-S14 Figs in S1 File, for a graphical representation of the data). The tabulated results are shown by ozone fumigation condition (i.e., concentration and RH level), contact time, and material. Overall, decontamination efficacy was evaluated for 252 cases (six materials × seven fumigation conditions × three contact times × two microorganism species). Ozone gas target concentrations ranged from 7,000 to 12,000 ppm, coupled with contact times ranging from 4–12 h.

**Table 6:** Inactivation (Log reduction) of *B*. *anthracis* spores.

Target Test Conditions, Ozone, %RH	Contact Time, h	Material Type					
		Glass	Wood	Carpet	Laminate	Metal Ductwork	Wallboard Paper
7,000 ppm, 75% RH (Test 2)	4	1.86 ± 0.39	2.53 ± 0.31	2.06 ± 0.23	1.51 ± 0.08	1.48 ± 0.16	5.21 ± 0.46
	6	2.33 ± 0.47	3.35 ± 1.59	3.03 ± 0.36	1.65 ± 0.08	1.44 ± 0.23	6.97 ± 0.91
	8	2.33 ± 0.52	2.95 ± 0.7	4.38 ± 1.07	1.58 ± 0.30	1.70 ± 0.08	6.34 ± 1.36
7,000 ppm, 85% RH (Test 1)	4	4.39 ± 1.29	4.13 ± 1.36	4.10 ± 0.86	3.11 ± 0.16	2.33 ± 0.17	**7.60** ± **0.10**
	6	2.71 ± 0.39	5.04 ± 1.35	6.13 ± 1.46	2.79 ± 0.17	1.98 ± 0.35	6.87 ± 0.88
	8	6.25 ± 1.53	**6.70** ± **0.11**	**7.82** ± **0.08**	4.99 ± 1.35	2.95 ± 0.72	**7.60** ± **0.10**
9,000 ppm, 75% RH (Test 4)	4	1.72 ± 0.24	2.31 ± 0.52	2.33 ± 0.19	1.27 ± 0.06	1.55 ± 0.20	3.65 ± 0.69
	6	1.70 ± 0.14	2.21 ± 0.24	3.16 ± 0.18	1.27 ± 0.06	1.40 ± 0.18	5.32 ± 1.79
	8	1.88 ± 0.37	2.56 ± 0.55	4.29 ± 0.76	1.49 ± 1.40	1.40 ± 0.4	5.42 ± 1.72
9,000 ppm, 85% RH (Test 3)	4	2.64 ± 0.31	6.01 ± 1.31	4.31 ± 0.16	2.21 ± 0.30	1.79 ± 0.16	7.17 ± 0.96
	6	3.26 ± 0.97	6.31 ± 0.72	**7.93** ± **0.06**	3.76 ± 0.28	2.17 ± 0.37	**7.66** ± **0.09**
	8	3.92 ± 1.95	**6.68 ± 0.10**	**7.93** ± **0.06**	3.10 ± 0.28	2.28 ± 0.15	**7.66** ± **0.09**
9,000 ppm, 85% RH (Test 5)	6	4.81 ± 1.67	6.34 ± 0.72	**7.90** ± **0.07**	2.75 ± 0.13	2.81 ± 0.41	**7.74** ± **0.05**
	9	5.68 ± 1.47	**6.71** ± **0.08**	**7.90** ± **0.07**	3.78 ± 0.72	3.90 ± 0.19	**7.74** ± **0.05**
	12	7.11 ± 0.99	**6.71** ± **0.08**	**7.90** ± **0.07**	2.74 ± 0.08	2.55 ± 0.40	**7.74** ± **0.05**
9,800 ppm, 85% RH (Test 6)	6	6.61 ± 1.42	**6.90** ± **0.27**	**7.92** ± **0.08**	3.52 ± 0.17	3.34 ± 0.48	**7.84** ± **0.48**
	9	7.33 ± 0.74	**6.90** ± **0.27**	**7.92** ± **0.08**	3.35 ± 0.3	4.00 ± 0.75	**7.84** ± **0.48**
	12	6.95 ± 1.48	6.60 ± 0.65	**7.92** ± **0.08**	5.39 ± 1.11	4.97 ± 0.51	**7.84** ± **0.48**
12,000 ppm, 85% RH (Test 7)	6	6.52 ± 1.03	**7.04** ± **0.46**	6.21 ± 1.50	5.05 ± 0.48	5.88 ± 0.98	**7.49** ± **0.03**
	9	6.69± 0.89	6.74 ± 0.75	**7.97** ± **0.03**	6.93 ± 1.07	5.79 ± 1.03	7.19 ± 0.60
	12	7.66 ± 0.41	**7.04** ± **0.46**	**7.97** ± **0.03**	5.82 ± 1.16	6.50 ± 1.00	**7.49** ± **0.03**

Efficacy data are expressed as mean ± SD log reduction. Results in bold signify all five coupons of material were completely decontaminated, i.e., no spores were detected on any of the five replicate coupons.

**Table 7:** Inactivation (Log reduction) of *B*. *subtilis*.

Target Test Condition, Ozone, %RH	Contact Time, h	Material Type					
		Glass	Wood	Carpet	Laminate	Metal Ductwork	Wallboard Paper
7,000 ppm, 75% RH (Test 2)	4	1.18 ± 0.22	0.38 ± 0.21	0.83 ± 0.24	1.01 ± 0.06	0.39 ± 0.42	3.07 ± 0.49
	6	1.88 ± 0.35	1.71 ± 2.17	0.87 ± 0.21	1.25 ± 0.36	0.44 ± 0.42	5.81 ± 1.56
	8	2.42 ± 0.11	0.42 ± 0.25	1.60 ± 0.23	1.53 ± 0.06	0.55 ± 0.42	5.17 ± 1.50
7,000 ppm, 85% RH (Test 1)	4	4.51 ± 0.93	1.48 ± 0.44	1.72 ± 0.56	6.49 ± 1.37	2.42 ± 0.27	6.84 ± 0.76
	6	6.51 ± 2.19	4.00 ± 1.66	2.82 ± 0.99	6.57 ± 1.28	2.51 ± 0.23	**7.44 ± 0.20**
	8	7.60 ± 0.78	3.30 ± 0.87	2.01 ± 0.38	**8.02 ± 0.09**	3.45 ± 0.32	**7.44 ± 0.20**
9,000 ppm, 75% RH (Test 4)	4	2.77 ± 0.67	0.69 ± 0.24	1.87 ± 0.35	2.70 ± 0.17	1.71 ± 0.26	5.87 ± 1.55
	6	3.91 ± 0.35	0.78 ± 0.23	2.02 ± 0.37	2.50 ± 0.34	1.75 ± 0.26	4.08 ± 0.74
	8	6.28 ± 1.02	1.14 ± 0.22	2.32 ± 0.29	3.84 ± 0.44	2.80 ± 0.26	6.67 ± 0.86
9,000 ppm, 85% RH (Test 3)	4	2.84 ± 0.42	0.74 ± 0.23	2.06 ± 0.29	2.78 ± 0.09	1.23 ± 0.34	3.17 ± 0.99
	6	3.87 ± 0.30	1.04 ± 0.14	2.15 ± 0.25	2.48 ± 0.14	1.32 ± 0.21	5.30 ± 1.45
	8	4.60 ± 0.21	1.53 ± 0.51	2.89 ± 0.18	2.91 ± 0.13	2.15 ± 0.43	5.52 ± 1.22
9,000 ppm, 85% RH (Test 5)	6	6.53 ± 1.11	2.62 ± 0.49	3.84 ± 0.52	**7.59 ± 0.05**	7.17 ± 0.61	**6.7 ± 0.14**
	9	6.89 ± 1.04	3.41 ± 1.53	5.14 ± 0.65	**7.59 ± 0.05**	6.86 ± 0.74	**6.75 ± 0.14**
	12	7.11 ± 0.61	5.87 ± 0.60	5.99 ± 1.56	7.29 ± 0.60	**7.47 ± 0.13**	**6.75 ± 0.14**
9,800 ppm, 85% RH (Test 6)	6	5.68 ± 0.94	4.54 ± 1.61	4.35 ± 0.47	7.51 ± 0.60	6.17 ± 0.77	**6.87 ± 0.14**
	9	6.59 ± 1.10	3.64 ± 1.58	5.34 ± 0.54	**7.82 ± 0.08**	**7.65 ± 0.15**	**6.87 ± 0.14**
	12	7.08 ± 0.73	5.70 ± 1.34	6.69 ± 1.29	**7.82 ± 0.08**	6.31 ± 0.69	**6.87 ± 0.14**
12,000 ppm, 85% RH (Test 7)	6	6.08 ± 1.26	2.80 ± 0.19	3.69 ± 0.14	**7.80 ± 0.07**	4.69 ± 0.29	**6.57 ± 0.06**
	9	7.30 ± 0.60	3.77 ± 0.95	4.05 ± 0.47	**7.80 ± 0.07**	6.42 ± 1.12	**6.57 ± 0.06**
	12	**7.60 ± 0.08**	3.13 ± 0.35	4.72 ± 0.65	**7.80 ± 0.07**	6.26 ± 1.39	**6.57 ± 0.06**

Efficacy data are expressed as mean ± SD log reduction. Results in bold signify all five coupons of material were completely decontaminated, i.e., no spores were detected on any of the five replicate coupons.

Overall, efficacy ranged from a low of 0.39 LR (Test 2, with galvanized metal ductwork, *B*. *subtilis*, 7000 ppm, 4 h, 75% RH) to complete inactivation (≥7.9 LR in some cases, depending on the positive control recovery). The LR results shown in bold in Tables 6 and 7 signify a test condition and material that resulted in complete inactivation, i.e., there were no spores recovered from any of the five replicate test coupons. There were several conditions (>50) in which this occurred, and complete inactivation tended to happen more frequently at the higher RH, at higher ozone concentrations, and longer contact times, as expected, although we caveat that there were several ozone fumigation conditions in which increasing contact time offered no increase in efficacy; see, for example, the tests at 7,000 and 9,000 ppm, at the lower 75% RH level, for both species. At the most robust decontamination condition of 12,000 ppm ozone and 85% RH, all but four (out of 18) test material/contact time combinations resulted in effective decontamination (≥ 6 LR) for *B*. *anthracis*.

There are few data from the literature with which to compare our efficacy results due to differences in test methodology, microorganisms, test materials, and ozone fumigation conditions. Nevertheless, we report the literature that may be somewhat relevant or comparable to our tests. For example, Aydogan and Gurol [12] showed improvement in efficacy against *B*. *subtilis* spores with increasing contact time, ozone concentration, and RH (consistent with our study), although their testing utilized a maximum ozone concentration of approximately 5,000 ppm and a maximum contact time of only four h, and never demonstrated efficacy ≥ 4 LR.

Only a few studies in the literature report ozone gas achieving ≥ 6 LR of bacterial spores except when using relatively high concentrations, consistent with our findings. As an example, Buranov et al.[22] in a conference proceeding reported that decontamination efficacy > 6 LR with an avirulent strain of *B*. *anthracis* was achieved with ozone concentrations of ~ 10,000 ppm, consistent with the present study. Currier et al.[13] demonstrated ≥ 6 LR against spores of *B*. *globigii* on glass coupons when using 9,000 ppm ozone with only a 1 h contact time. These results are comparable to our testing, in which ≥ 6 LR was achieved for *B*. *subtilis* on glass at 9,000 ppm, 75% RH; however, an 8 h contact time was needed. The demonstration of effective decontamination only after 1 h by Currier et al.^11^ may be due to the spores being preconditioned by exposure to elevated RH levels for 15 h prior to fumigation with ozone. In contrast, Sharma et al.[6] tested ozone gas at a low level of 25 ppm against spores of *C*. *difficile* and *B*. *cereus*, but the populations of these bacteria were insufficiently high (up to a maximum of 4 log CFU) to demonstrate ≥ 6 LR.

#### Effect of material

Six materials were included in the present study to assess their effect on decontamination efficacy and included both porous (carpet, wood) and nonporous (glass, laminate, galvanized metal, and wallboard paper) materials. Overall, ozone gas was effective in inactivating *B*. *anthracis* spores on all materials under at least one test condition, and for *B*. *subtilis*, ozone gas was effective under at least one test condition for all materials except wood. For some materials, such as wood, carpet, and wallboard paper, effective decontamination was achieved for *B*. *anthracis* with the lowest ozone concentration tested (7,000 ppm), while materials such as laminate and metal ductwork required 12,000 ppm to achieve ≥ 6 LR of *B*. *anthracis*. Further, the effect of material on efficacy varied by the species. For example, in terms of the number of successful decontamination events (≥ 6 LR) or lack thereof, spores of *B*. *anthracis* were the most difficult to inactivate when associated with the laminate, metal ductwork, and glass materials, while *B*. *subtilis* was more difficult to inactivate when associated with wood and carpet. It is possible that this difference may be attributed to structural or molecular differences between the two species. For instance, the spore outer coat is comprised of different proteins for both species, and *B*. *anthracis* spores possess an exposporium, whereas *B*. *subtilis* spores do not.[25] For both species, the painted wallboard paper material was effectively decontaminated the most frequently (62% of the tests for *B*. *anthracis*, and 81% of the tests for *B*. *subtilis*) compared to the other materials. Painted wallboard paper and other organic materials (carpet, wood) perhaps generate reactive oxygen species (ROS) upon exposure (and reaction) to ozone gas, which may explain why efficacy is higher for these materials (further discussed below).

The Aydogan and Gurol study[12] was the only study we found that used somewhat comparable test materials and demonstrated that wood and carpet were more difficult to decontaminate compared to materials such as glass and floor tile. This finding is consistent with our observations relative to the effect of material on ozone gas efficacy for inactivation of *B*. *subtilis*. However, for *B*. *anthracis*, we found that effective ozone decontamination occurred more often on wood and carpet compared to glass, laminate and metal ductwork. It is unclear why this difference in results between the two species occurred. Generally, inorganic, nonporous materials such as glass, laminate, or galvanized metal are typically easier to effectively decontaminate compared to porous and organic materials such as carpet and wood, especially when using oxidation-based sporicides such as ozone.[3] The observation that *B*. *anthracis* spores were relatively more difficult to inactivate with ozone gas on glass, galvanized metal and laminate indicates the possibility of a different inactivation mechanism involved. Ozone gas is known to react with organic matter to produce ROS (which may include hydroxyl and peroxyl radicals, and peroxides[34–36]), and these ROS may be sporicidal themselves. Ozone gas itself is considered an ROS; ROS generated from non-thermal plasmas have been shown to be sporicidal[37]. Further research is recommended to better elucidate the potential different inactivation mechanisms for the two species.

#### Effect of RH

In the tests in which the lower RH of 75% was utilized (Tests 1 and 3), there were only two occurrences (out of 72, or ~3%) in which effective decontamination was achieved (*B*. *subtilis*; wallboard paper and glass; at 9,000 ppm). In contrast, for Tests 2 and 4, which were conducted under the same conditions as Tests 1 and 3 except at the higher RH of 85%, 32% of the results demonstrated effective decontamination (Refer to the Supporting Information for a graphical representation of the data). In these graphs, we can observe that the inactivation efficacy for *B*. *anthracis* was higher at 85% RH than at 75% for most of the comparisons, but the difference in efficacy for the two RH levels was less pronounced on the glass and metal materials. The general result showing improved efficacy with increased RH is consistent with other studies[12, 14–16]. Comparable to our findings, Ishizaki et al.[16] demonstrated an approximately 3 log improvement in the inactivation of *B*. *cereus* and *B*. *subtilis* spores on filter paper when RH was increased from 70 to 80%. The statistical model also confirms that there is strong evidence that RH does affect decontamination efficacy, with a p-value < 0.0001.

#### Effect of species

As discussed above, the effect of microorganisms on decontamination efficacy with ozone gas varied somewhat by the material onto which the spore population was deposited. Overall, out of the 126 test conditions for each species (six materials × three contact times × seven fumigation conditions), there were 53 occurrences in which decontamination was effective against *B*. *anthracis*, and 45 occurrences in which decontamination was effective against *B*. *subtilis*. This global comparison of the two species indicates that *B*. *subtilis* may be slightly more resistant to ozone gas inactivation compared to *B*. *anthracis*. Overall, the results from the statistical analysis indicate there was no significant difference between the two species, with a p-value of 0.32. But when accounting for material interactions in the model, the species does have a significant effect (p <0.0001). This observed similarity or increase in resistance of *B*. *subtilis* to ozone gas as compared to *B*. *anthracis* is consistent with the literature in which the resistance of *B*. *subtilis* or *B*. *atrophaeus* is comparable to *B*. *anthracis* with the use of several other decontaminants[3]. We believe our study is the first to make the direct comparison between the two species relative to their resistance to ozone gas inactivation and supports the use of *B*. *subtilis* as a surrogate for *B*. *anthracis* for this decontaminant. Further research is recommended to demonstrate the ozone gas decontamination technology at a larger scale.

## Supporting information

S1 File Decontamination efficacy results plotted by microorganism, material, and by ozone gas fumigation condition. (DOCX)
