# Supplementary material for: The use of ozone gas for the inactivation of Bacillus anthracis and Bacillus subtilis spores on building materials
Source: PLoS One. 2020 May 21;15(5):e0233291. doi: 10.1371/journal.pone.0233291 (PMC7241793; doi:10.1371/journal.pone.0233291)
Supplement: S1 File — (DOCX) [file pone.0233291.s001.docx]

**Decontamination Efficacy Results for *B. anthracis* Ames**

(WBP=wallboard paper; * = complete inactivation)


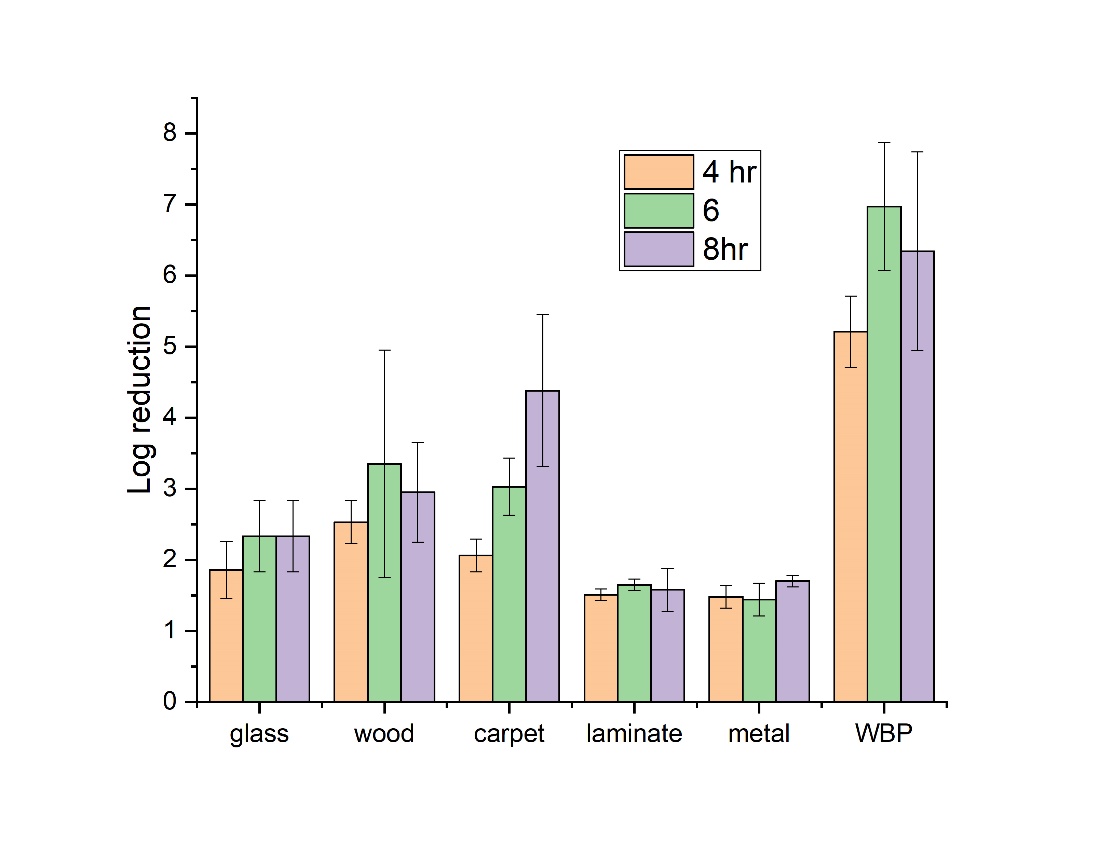


**Figure S1*. B. anthracis* 7000 ppm ozone, 75% RH**


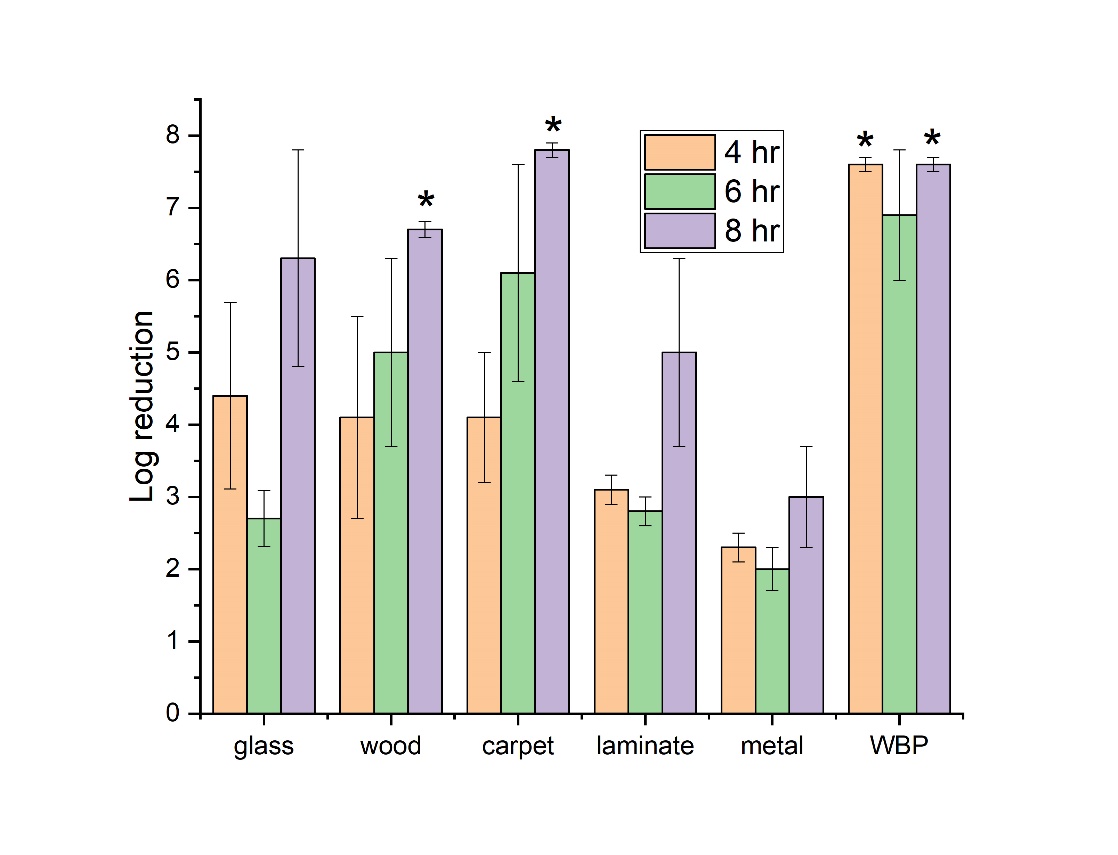


**Figure S2. *B. anthracis* 7000 ppm ozone 85% RH**


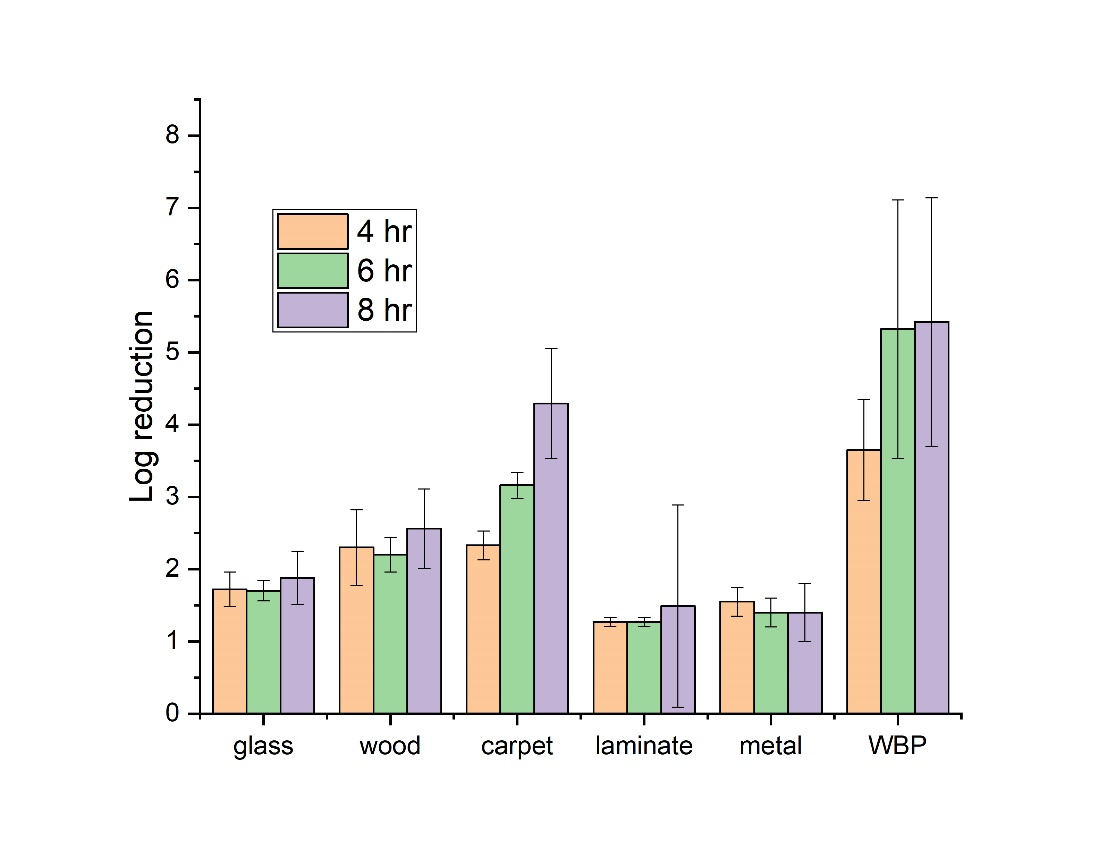


**Figure S3. *B. anthracis* 9000 ppm ozone, 75% RH**


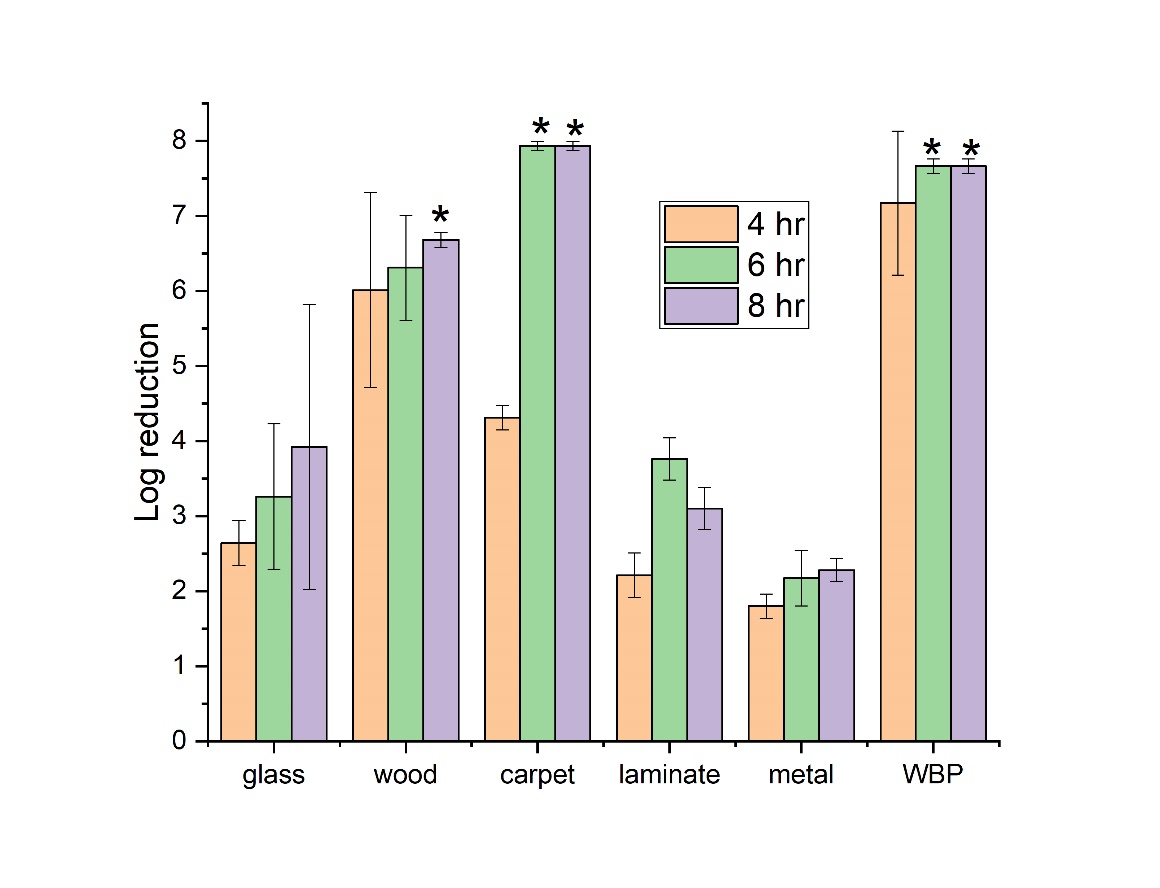


**Figure S4. *B. anthracis* 9000 ppm ozone, 85% RH**


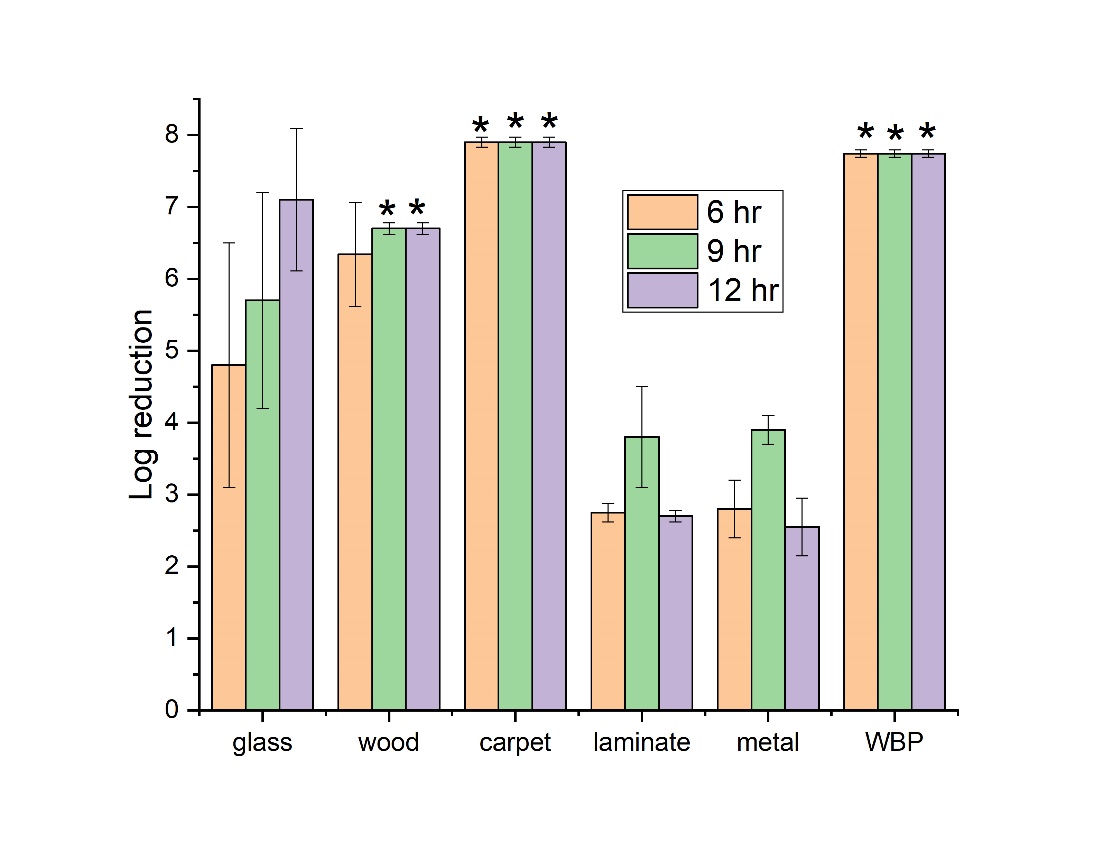


**Figure S5. *B. anthracis* 9000 ppm, 85% RH**


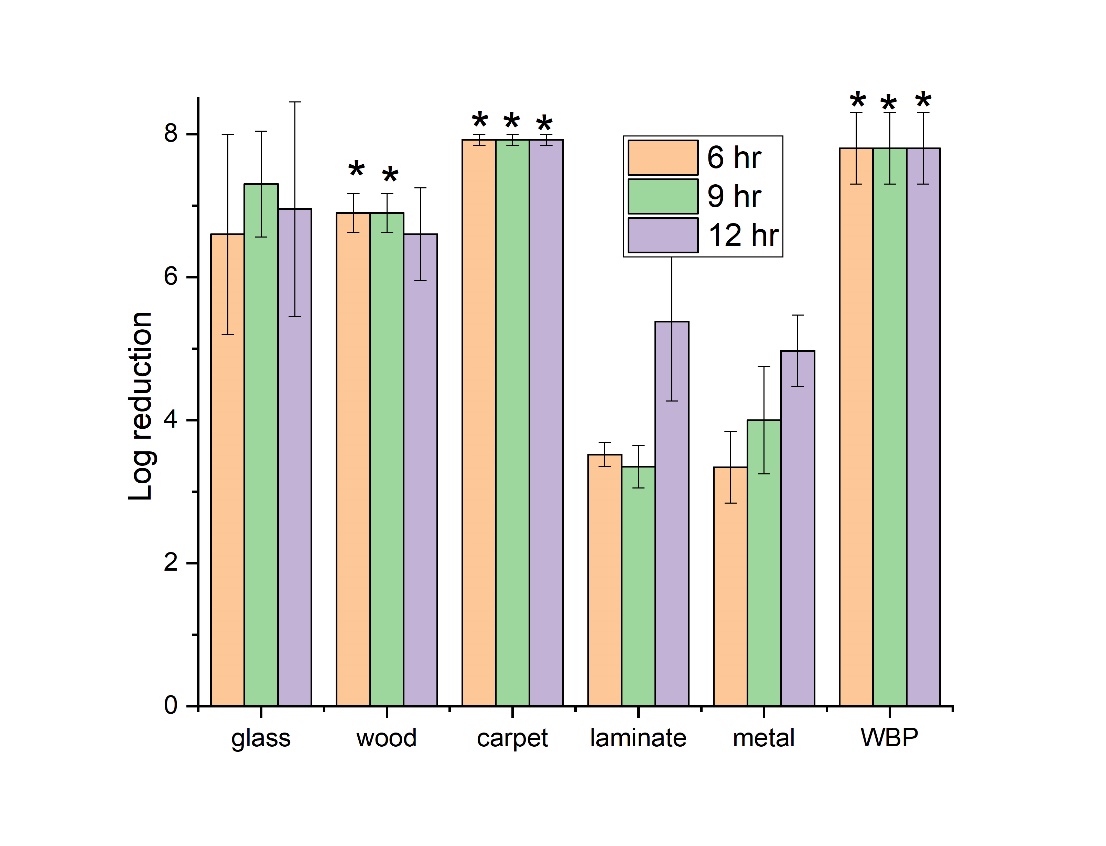


**Figure S6. *B. anthracis* 9800 ppm ozone, 85% RH**


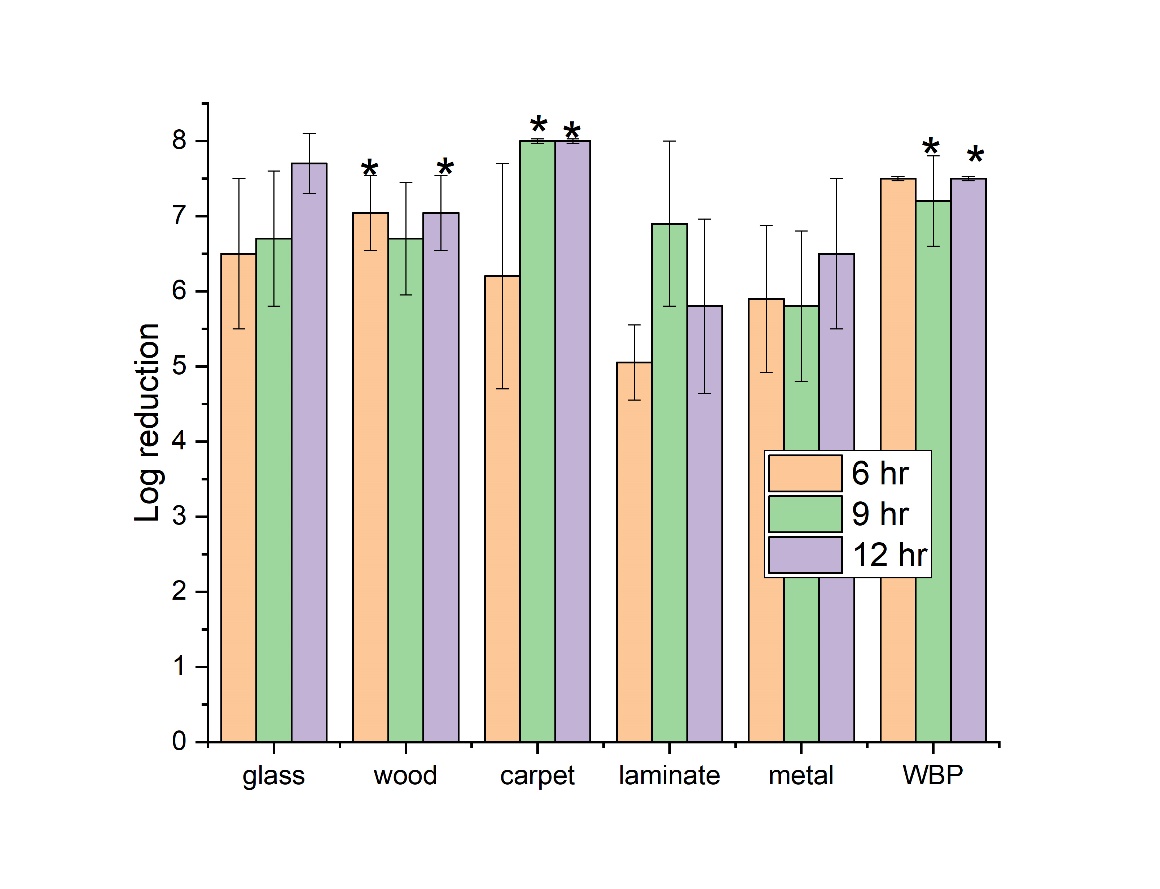


**Figure S7. *B. anthracis*, 12000 ppm ozone, 85% RH**

**Decontamination Efficacy Results for *B. subtilis***

(WBP=wallboard paper; *= complete inactivation)


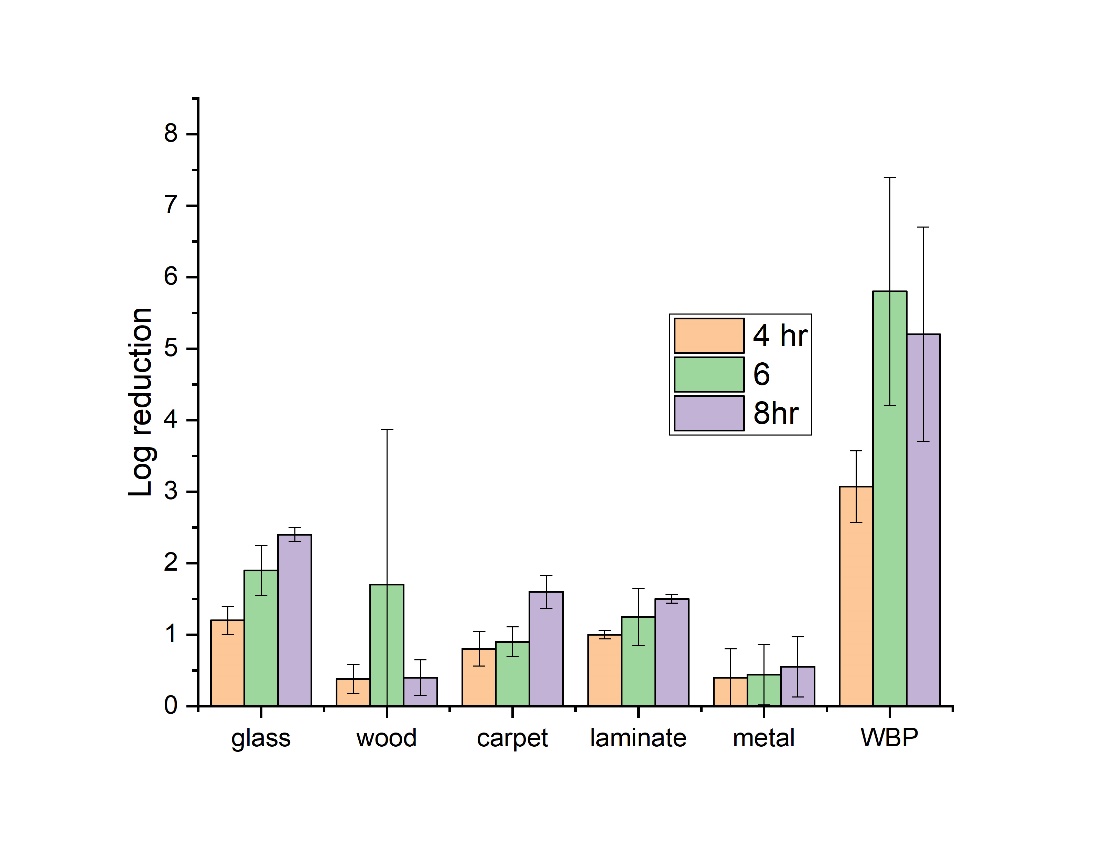


**Figure S8. *B. subtilis*, 7000 ppm ozone, 75% RH**


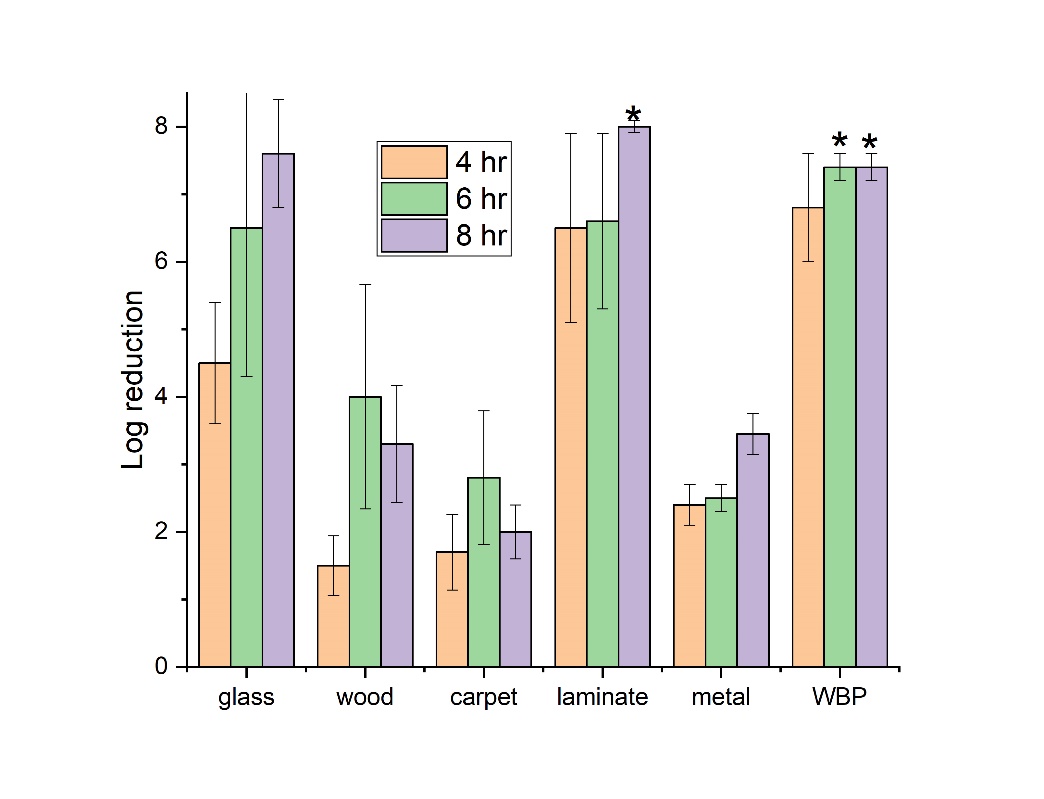


**Figure S9. *B. subtilis*, 7000 ppm ozone, 85% RH**


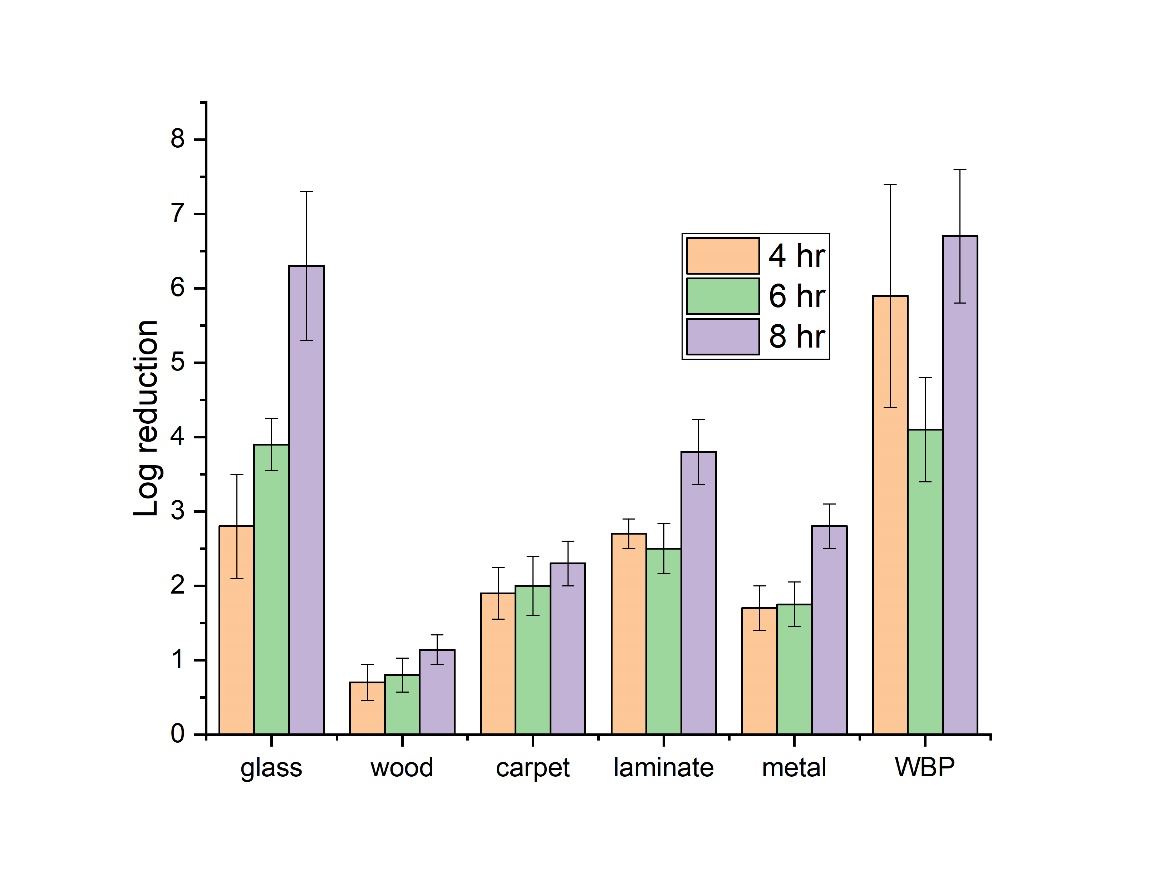


**Figure S10. *B. subtilis*, 9000 ppm ozone, 75% RH**


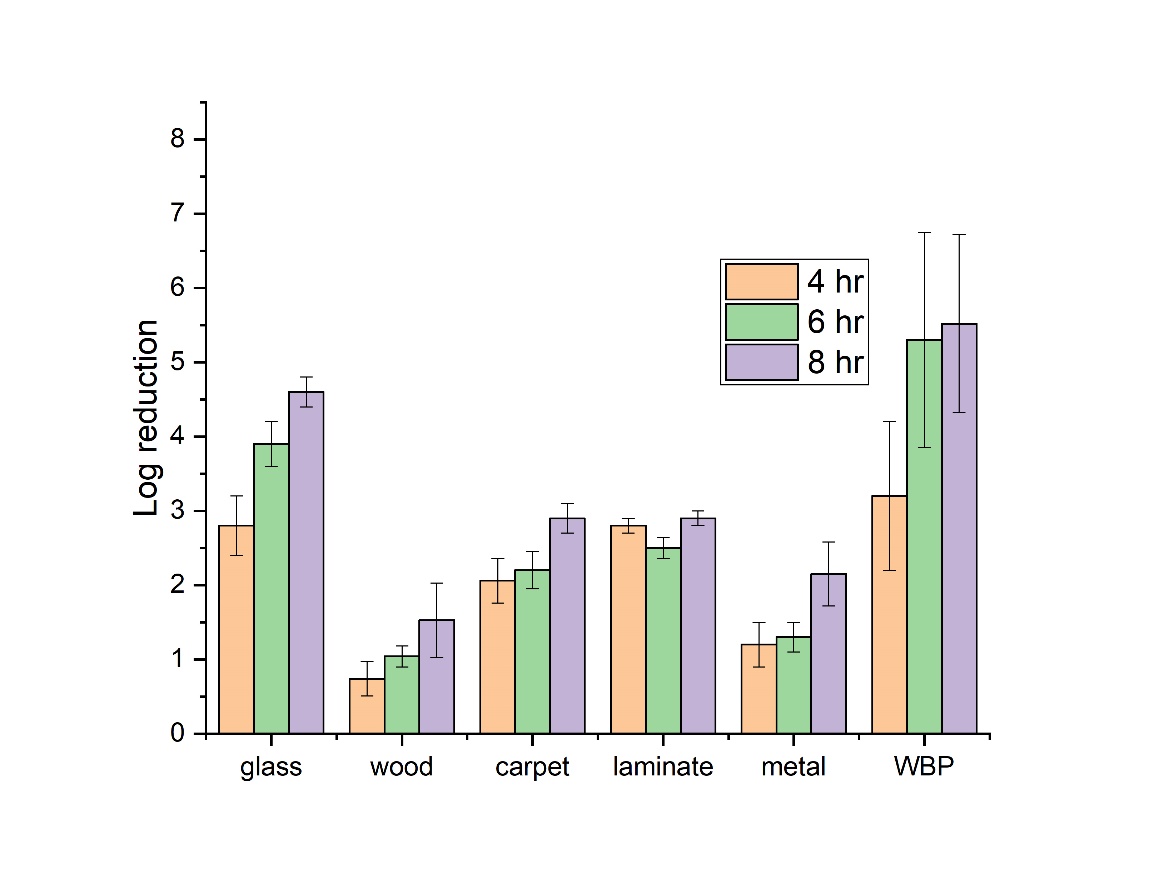


**Figure S11. *B. subtilis*, 9000 ppm ozone, 85% RH**


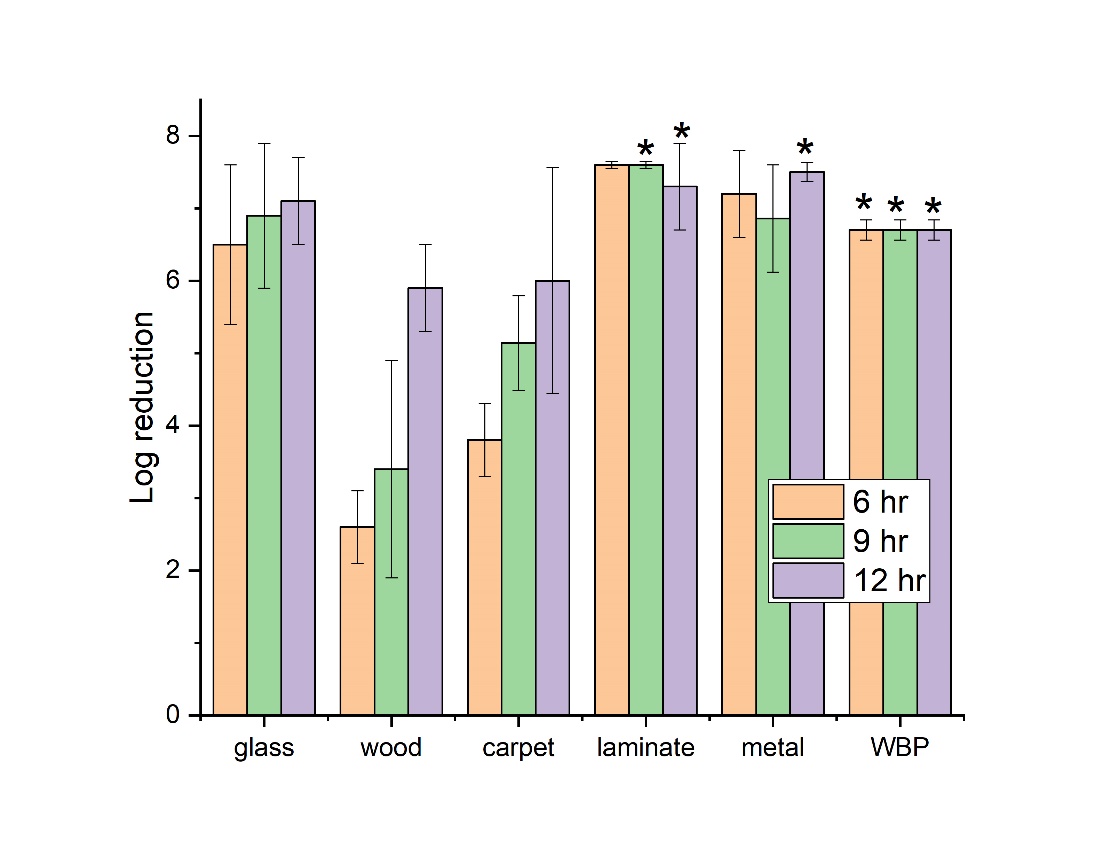


**Figure S12. *B. subtilis* 9000 ppm ozone, 85% RH**


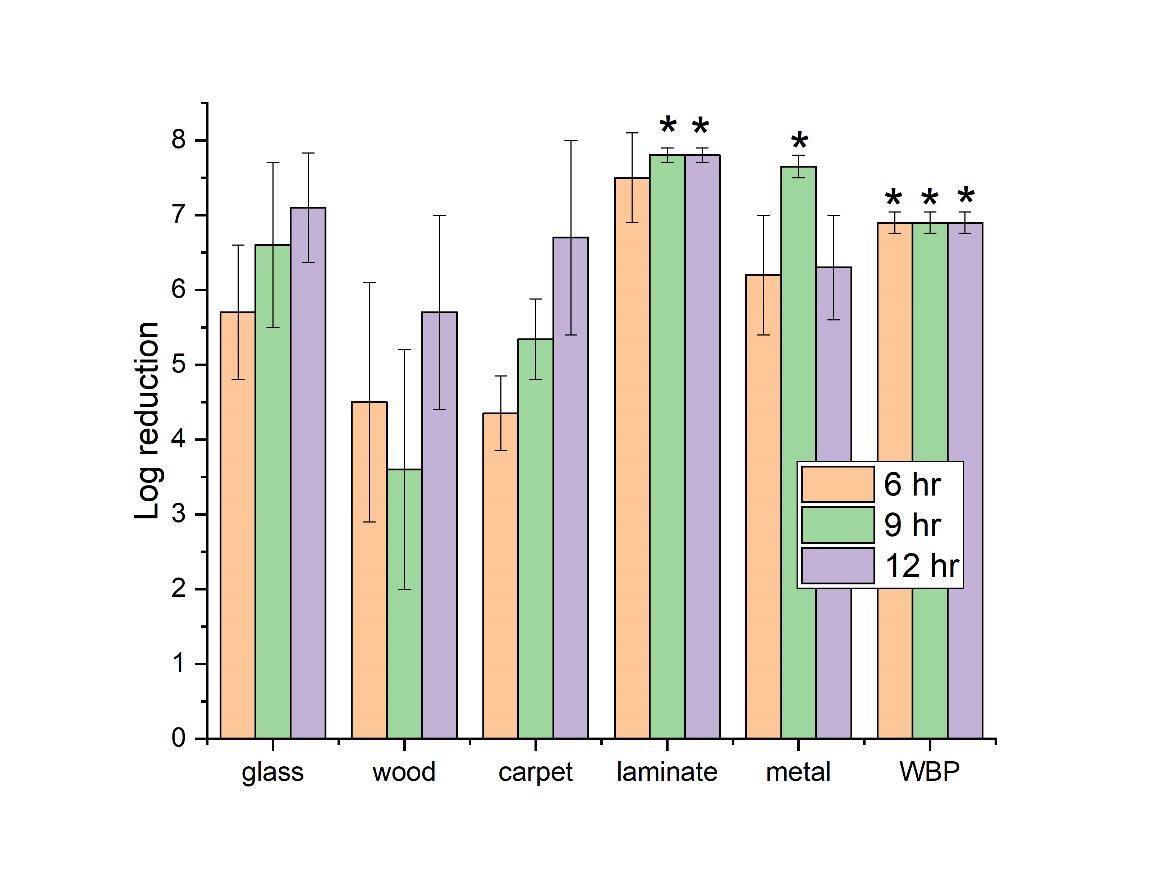


**Figure S13. *B. subtilis* 9800 ppm ozone, 85% RH**


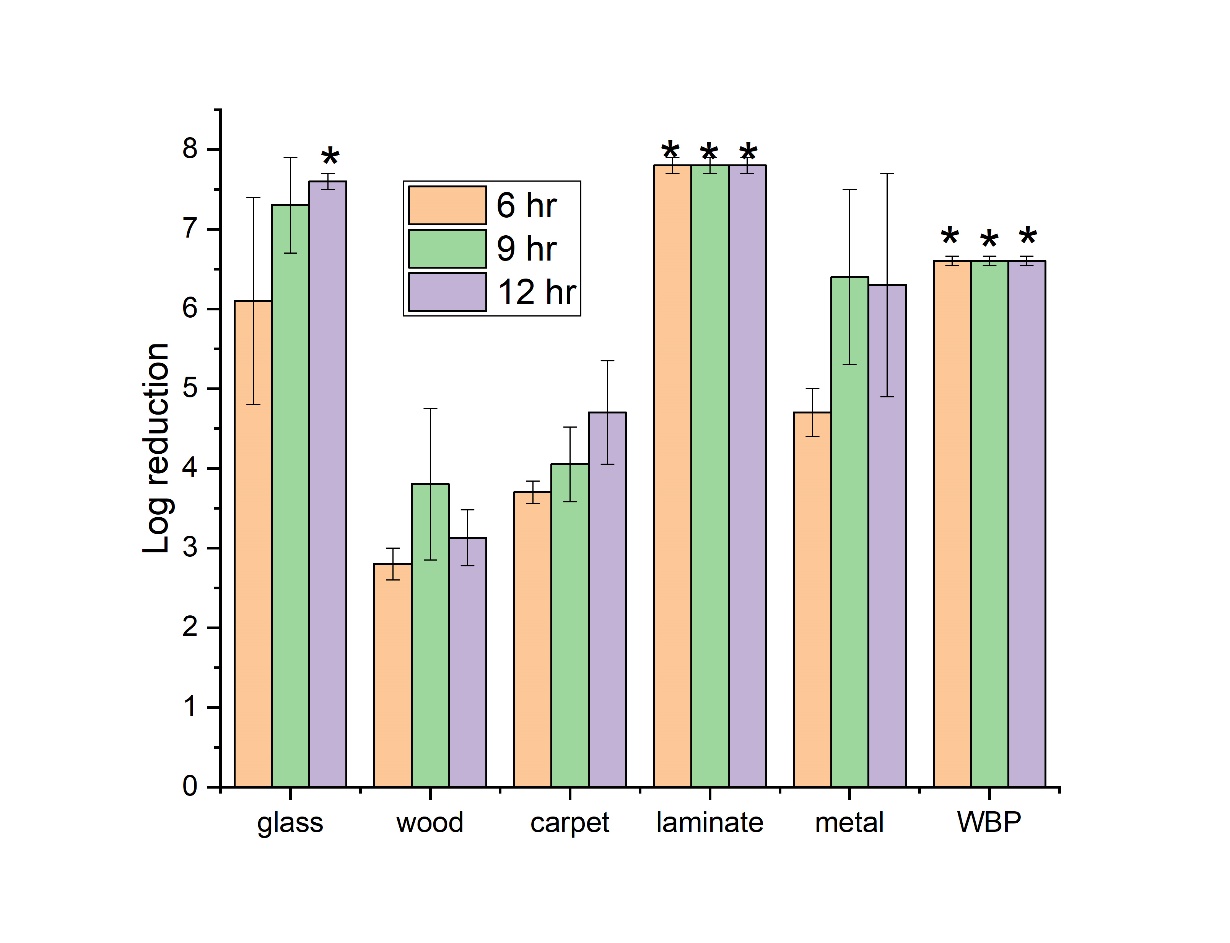


**Figure S14. *B. subtilis* 12000 ppm ozone, 85% RH**
